# Facial Self-Touching and the Propagation of COVID-19: The Role of Gloves in the Dental Practice

**DOI:** 10.3390/ijerph18136983

**Published:** 2021-06-29

**Authors:** María Carrillo-Díaz, Laura Lacomba-Trejo, Martín Romero-Maroto, María José González-Olmo

**Affiliations:** 1 Department of Orthodontics and Pediatric Dentistry, Rey Juan Carlos University, Alcorcón, 28922 Madrid, Spain; maria.carrillo@urjc.es (M.C.-D.); martin.romero@urjc.es (M.R.-M.); 2 Department of Personality, Assessment and Psychological Treatments, Faculty of Psychology, University of Valencia, Av. Blasco Ibáñez, 21, 46010 Valencia, Spain; laura.lacomba@uv.es

**Keywords:** COVID-19, SARS–CoV–2, touch, anxiety, high risk, preventive measures

## Abstract

Background: Despite facial self–touching being a possible source of transmission of SARS–Co–V–2 its role in dental practice has not been studied. Factors such as anxiety symptoms or threat perception of COVID-19 may increase the possibility of contagion. The objective was to compare the impact of control measures, such as gloves or signs in the reduction in facial self–touching. Methods: An intra–subject design was undertaken with 150 adults. The patients’ movements in the waiting room were monitored with Microsoft Kinect software on three occasions: without any control measures, using plastic gloves or using advisory signs against self–touching. Additionally, the participants completed the sub–scale of STAI (State–Anxiety) and the BIP–Q5 (Brief Illness Perception Questionnaire); their blood pressure and heart rate were recorded. Results: The lowest incidence of facial self–touching occurred in the experimental situation in which gloves were introduced. The subjects with elevated anxiety symptoms realized more facial self–touching regardless of the control measures. However, the threat perception of COVID-19 is associated negatively with facial self–touching. Conclusions: The use of gloves is a useful control measure in the reduction in facial touching. However, people with anxiety symptoms regardless of whether they have greater threat perception for COVID-19 exhibit more facial touching.

## 1. Introduction

At the end of 2019 and the beginning of 2020 China announced the appearance of a new coronavirus. This virus known as SARS–CoV–2 spread rapidly throughout the world, resulting in COVID-19 being declared a pandemic in March [1,2].

With the purpose of reducing infection and without effective treatment available, governments throughout the world declared varying measures of isolation and restriction [3,4].

Since the global pandemic was officially declared by the World Health Organization, Madrid has become one of the main foci of COVID-19 in Europe. By 13 December 2020 there were 304,616 cases of infection recorded and 24,860 deaths in Madrid [5].

Due to the fact that the transmission of COVID-19 can occur through direct contact with people who have the virus (symptomatic or asymptomatic), aerosols or contaminated inanimate objects, the dental services were classified as potential risk locations for the transmission of the virus [6,7].

Although vaccination against COVID-19 has begun, to date no cure has been found and in fact recently a new, potentially more virulent strain of the virus has been identified [8,9]. For this reason, the regular washing of hands and the use of facial masks as well as the avoidance of facial self–touching is important. One of the main channels of contagion is the mucous membranes, including eyes, nose and mouth [7,10,11]. In view of the above, the use of gloves has been used in the general population as a preventive behavior, although hand washing is considered the most efficient [12].

However, as far as is known, no studies currently exist that have evaluated the using of gloves or warning signs or any other control measures in the general population that may influence behaviour and inhibit the spread of the virus.

Nevertheless, factors such as the experiencing of anxiety symptoms may lead to a greater incidence of self–touching, and as proposed in The Theory of Lang (1968), anxiety has a threefold response system (cognitive, behavioural and physiological) [13,14]. 

The manifestations of anxiety can include trembling, taquicardia, increase in blood pressure, as well as movements, which may include facial touching [15]. In this context, the thoughts that a person has about a stress situation can mediate the appearance of stress symptoms and result in a corresponding increase in physiological and physical movement expression [16].

Taking into account that odontology clinics are considered high risk locations for COVID-19, the aim of the study was to examine whether the use of control measures (gloves or signs) could reduce facial self–contacts.

## 2. Materials and Methods

### 2.1. Design Type

This intra–subject design research was carried out in Spain from 12 October to 11 December, 2020 during the second wave of the pandemic. 

### 2.2. Data Collection

The participants comprised 150 adult patients (over 18 years old) who attended four dental clinics in Madrid. All the patients were regular patients of the clinic, who had an appointment for an orthodontic check–up. The study’s objectives and nature were explained beforehand by telephone, and patients who agreed to participate in the study were enrolled.

Patients were asked to come to the dental clinic at the agreed–upon time (to avoid unnecessary waiting) unaccompanied and with a mask. One patient was scheduled per 30 min to avoid person–to–person contact. Upon arriving at the clinic, patients were asked to rub their hands with a hydroalcoholic gel for 20 s and to put on shoe covers, as established by the protocol for preventing COVID-19 transmission in Spain [17].

The patients were informed that their behaviour in the waiting room would be observed for a study. To blind them to the study, they were not informed about which behaviours were under observation to minimize the potential for behavioural changes due to being observed. After entering the waiting room, participants signed the informed consent form and were instructed to sit down. Their pulse and blood pressure were taken after sitting for five minutes after which the chair they had sat in was disinfected.

All participants gave prior consent to having their movements monitored. The movement monitoring time of 30 min was the minimum time for the preparation and disinfection of the dental chair area. From an ethical point of view and considering the risk involved, it was agreed that patients stay the minimum time possible. During their time there, 7 min were allowed for the measuring of blood pressure and pulse, 15 min for the monitoring of facial self–touching (eyes and mask) and the remaining 8 min for questions and answers. All participants were subjected to the same conditions and in the same order.

These participants generally have orthodontic checks every three weeks. At each appointment during a period of three months they were subjected to three experiments in which the incidence of self–touching was monitored, including different elements with the aim of observing if these measures reduce facial self–touching.

At the first appointment, facial self–touching was monitored without including any measure of control (Experiment 1). At the second appointment, plastic disposable gloves were worn (Experiment 2) and at the third appointment advisory signs, which remind the patients to avoid touching the face, were placed on the walls (Experiment 3).

After this recording, participants filled out self–administered instruments using Microsoft forms to avoid contact with the paper. The questionnaire’s link was sent via e–mail or WhatsApp to their mobile device, and they completed it after the movement monitoring time in the waiting room.

This research is supported by the King Juan Carlos University Ethics and Research Committee (Registration number: 0103202006520).

### 2.3. Instruments:

A questionnaire was developed to evaluate socio–demographic variables of age, gender, educational level (uneducated, primary, secondary or university degree). In addition, data were collected on their previous psychological problems (yes/no).

The Microsoft Kinect was used to evaluate the detection and counting of movement patterns [18,19,20,21,22,23].

Anxiety symptomatology was evaluated as a trait using the trait anxiety subscale of the State–Trait Anxiety Inventory (STAI). The STAI is a self–report questionnaire comprising a state–anxiety subscale (how one feels in a particular time or situation) [24].

To evaluate perceived threat from COVID-19 the Brief Illness Perception Questionnaire version BIP–Q5 was used [25].

The heart rate and the blood pressure (systolic [mmHg] and diastolic [mmHg]) were measured by a member of the research team with a high–precision mercury sphygmomanometer. The blood pressure was taken sitting down.

The description of the instruments is attached in the online Appendix A. https://github.com/mariajosegonzalez123/online-appendix.git (accessed on 29 June 2021)

### 2.4. Statistical Analysis

The study presents a longitudinal descriptive study considering the variables de–scribed in the previous section. A statistical analysis was performed using SPSS v26 (SPSS Inc., Chicago, IL, USA). The data analysis included descriptive statistics and the Kolmogorov–Smirnov test to evaluate the assumption of normality, which was confirmed. To know possible differences, t–tests and ANOVA were performed. Scheffé (if equal variance is assumed) and Games–Howell (if not) post hoc tests were used, and effect sizes were calculated. For t of independent samples, a Cohen’s d was performed. According to Cohen (1988), small Cohen’s d values are ≈0.2, medium ones are ≈0.5, and high ones are ≈0.8. For the ANOVA test, partial eta squared was carried out. Cohen (1988) considers small effect size values to be ≈0.01, medium ones to be ≈0.06, and those large enough to be taken into account as ≈0.14 [26].

The relationships between variables were analyzed using Pearson’s correlations. As the self–touching in the three different experimental situations was correlated, the assumption of sphericity was examined using the Mauchly test. Finally, a procedure such as the Greenhouse–Geisser correction was used to address violation of sphericity.

Repeated measurement analysis was performed to examine variations between and within subjects with regard to facial self–touching in the different experimental situations. Bonferroni’s multiple comparison procedure was calculated to identify differences in facial self–touching in the three different experimental situations. Significance was set at *p* < 0.01.

A cut point of 39–40 has been suggested to detect clinically significant symptoms for the STAI–S scale, Low anxiety for STAI–S < 39, High Anxiety for STAI–S ≥ 39 [27]. Two cut points for BIPQ–5 were estimated based on the medium of 15 previous studies, low BIPQ–5 (<30), high BIPQ–5 (≥30).

## 3. Results

### 3.1. Sociodemographic Variables

The sample comprised 62 men and 88 women, with an age range of 20 to 47 years (29.91 ± 6.76). In terms of educational levels for the total sample, 45.3% had completed primary school, 30% had completed secondary school, and 24.7% had obtained a university degree.

Table 1 presents the descriptive data of the variables STAI–S, BIPQ–5, having suffered COVID-19, that some relative had suffered COVID-19, Self–touching/minute Experimental situation 1, Self–touching/minute Experimental situation 2, Self–touching/minute Experimental situation 3, Heart rate/minute, (systolic blood pressure and Diastolic blood pressure). Mean differences in sociodemographic factors were measured (age, gender and educational level) regarding the target variables. Statistically significant differences were only found concerning participants’ gender.

As shown in Table 2, women presented more state–anxiety (♀20.61 ± 14.80, ♂14.91 ± 11.88, *p* = 0.01). People with previous psychological problems (anxiety, depression) presented more state–anxiety (*p* < 0.01). Where statistically significant differences occurred, moderate/large effect sizes were observed in all comparisons.

### 3.2. Facial Self-Touching

Neither age nor educational level is associated with facial self–contacts.

An elevated perception of the threat of COVID-19 is associated with a statistically significant state–anxiety, as is the pulse and blood pressure. In experimental situations 1 and 3 this is also expressed by an increase in facial self–touching. An elevated level of self–touching is associated with state–anxiety, while major threat perception is associated negatively with facial self–touching, independent of the control measures. See Table 3.

**Figure 1:**
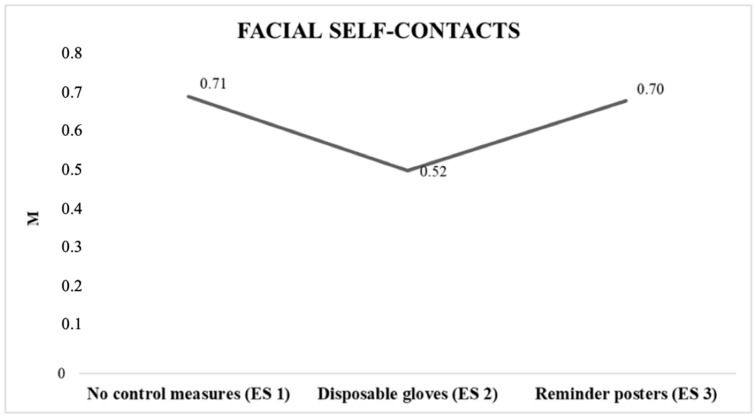
Average number of self–contacts per minute in the different experimental situations. Note: ES = experimental situation. ES 1 (no control measures), ES 2 (disposable gloves), ES 3 (reminder posters).

As there were differences in facial self–touching in the different experimental situations, a repeated measure analysis was conducted to determine the location of the differences. Repeated measurements of the ANOVA revealed that there were significant differences in facial self–touching for the three experimental situations F (1, 149) = 340.08, *p* < 0.01, η^2^ = 0.69. A minor frequency of self–touching was produced in experimental situation 2, in which the participants wore gloves, in experimental situation 2, (without any control measure) and the experimental situation 3 (with advisory signs) (Table 4, Figure 1).

**Figure 2:**
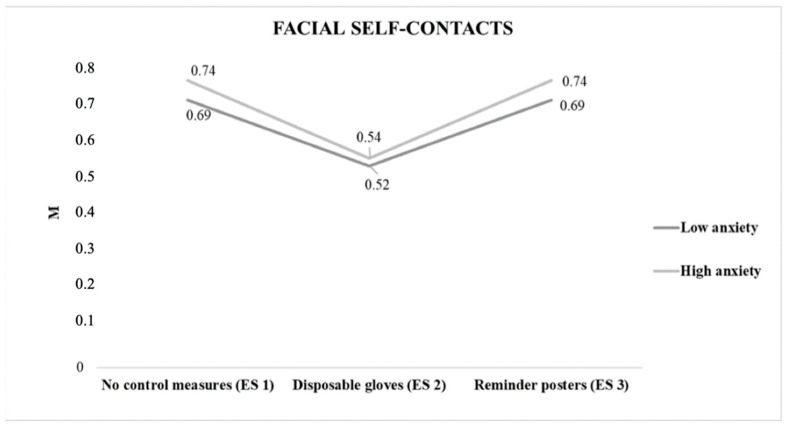
Average number of self–contacts per minute in different experimental situations according to anxiety levels. Note: ES = experimental situation. ES 1 (no control measures), ES 2 (disposable gloves), ES 3 (reminder posters).

Since the psychological variables could act as confounding variables, we proceeded to explore them. Cut points for STAI–S were estimated: more anxious subjects (≥39), less anxious subjects (<39). An effect of interaction was observed between the groups of anxiety and different situations (*p* < 0.01) (Figure 2).

**Figure 3:**
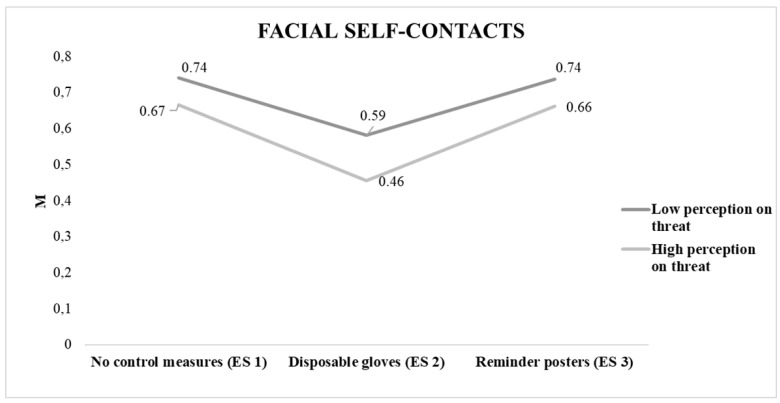
Average number of self–contacts per minute in different experimental situations according to perception on threat level. Note: ES 1 (no control measures), ES 2 (disposable gloves), ES 3 (reminder posters).

And as can be seen in Figure 3, intra–group differences were found (F (1,2) = 6.61, *p* < 0.01, η^2^ = 0.04, observed potency = 0.87) and inter–group (F (1,2) = 25.38, *p* < 0.01, η^2^ = 0.14, observed potency = 0.99).

As can be observed in Table 5, a multiple hierarchical regression was carried out to determine if the presence of anxiety symptoms (cognitive and physiological), as well as a major perception of COVID-19 increased the prediction of self–touching with gloves. The complete model of BIPQ–5, STAI–S and pulse, for predicting self–touching with gloves (Model 3) was statistically significant and predicted a total of 24.6% of the variance of facial self–contacts (F (3,146) = 17.22, *p* < 0.01).

The addition of BIPQ–5 to the prediction (Model 1) led to a statistically significant increase in R^2^ of 0.23, F (2,147) = 25.709, *p* < 0.01.

In this way a lesser incidence was observed of self–touching (with gloves), of anxiety symptoms expressed cognitively and physiologically as well as a lower threat perception of COVID-19, all of which increased the frequency of facial self–touching.

## 4. Discussion

While the pandemic of COVID-19 has posed a social and economic challenge it is especially serious in the sanitary field [28]. Without a cure for the virus, society must resort to control measures to avoid its propagation. It is understood that the use of masks and the washing of hands can contribute to the reduction in the transmission of COVID-19 [7,10]. However, until the present, studies do not exist that approach the effectiveness of other types of control measures that are believed to be efficient to differing degrees.

Therefore, the present study by means of an experiment in a real but controlled setting has evaluated the use of other control measures for facial self–touching such as gloves or advisory signs. Responding in this way to the main goal of our study, which is to analyze if the presence of control measures such as these can reduce the incidence of self–touching in odontological clinics as a measure of infection control.

It has been observed that the use of latex gloves is a useful measure for the reduction in facial self–touching in odontological clinics. In fact, in the experimental situation studied, a lesser incidence of facial self–touching was noted when reminder signs were introduced compared with when control measures were absent. This is especially important considering the possible relationship between facial self–touching and infection from SARS–CoV–2 [15].

The present study proposes to examine if variables such as anxiety symptoms (cognitive and physiological) and the threat perception of COVID-19 can be associated with facial self–touching, that is with the motor expression of anxiety symptoms. Our results show how in the experimental situation of wearing gloves, anxiety symptoms (cognitive and physiological) and a lesser threat perception increase the incidence of facial self–touching. As is suggested by the literature, different expressions of anxiety are associated together [13,14], suggesting that people with greater anxiety symptoms may be more vulnerable to infection as they experience a greater incidence of facial self–touching [29].

Despite there not being previous studies which associate greater threat perception of COVID-19 with facial self–touching, it is hoped that frequent facial self–touching as a repetitive unconscious behavioural function, which may help and regulate the emotion, is tempered by the very specific fear and perceived risk of infection related with COVID-19. Given the nature of the fear stimulus of contracting COVID-19 it is much more likely that a certain hyper–vigilance is developed against possible threats (germs) and “unclean” behaviour such as touching the face, forming an inverse relationship between these variables.

The present study also proposes to evaluate the differences in anxiety symptoms in relation to gender. Different components of anxiety symptoms are found to be associated together, nevertheless only state–anxiety is more present in women and people with previous mental health problems. This aspect is of special importance as other studies have indicated that women and people with mental health problems have been the groups most affected emotionally and mentally during this pandemic [30,31,32].

Despite the significant contributions of our study to the prevention of the expansion of COVID-19 in odontological clinics, the study is not exempt from limitations. Specifically, one of the main limitations of the analysis is that it does not permit the establishment of causal relationships. Additionally, an increase in the number of participants in future studies is recommended. However, until the present, there have been few intra–subject and longitudinal studies that have been undertaken during the pandemic and even less that have had this number of participants evaluated with subjective and objective measures.

Additionally, this design permits the reduction or elimination at the last moment of the individual differences attributed to the error in the variation. Added to the above it is expected that the order of presentation of the experimental situations will not modify the results because these situations are totally independent and would have no cause to alter the behaviour of the participants. However, in future research it would be recommended to randomize the order of presentation of the experimental situations in order to reduce the possible error caused by their order of presentation. As the current situation is particularly delicate and changeable, the researchers considered it acceptable that all participants were exposed to the same conditions in the same order.

This study displays data collected in a natural setting for the participants, which reduces the experimental reactivity but at the same time endeavours to control odd variables that could obscure the results. In addition, it evaluates the participants by means of subjective and objective procedures, which reduce the errors associated with social desirability.

This work is novel in relation to the resources employed and its results and is especially important in the odontological field, a high risk setting for contagion. The data indicate the importance of the use of gloves as a control measure for the reduction in facial self–touching and its relevance with regard to future contagion in odontological clinics. Despite the benefits found in this regard, we believe that there is a need for educational interventions to ensure the correct use of gloves. Of particular interest to reduce the risk of infection is the proper removal of gloves. Future research should investigate whether there is also an increased likelihood of touching the face once the gloves are removed.

It also indicates the importance of psychological variables associated with the in–crease in facial self–touching, noting how those people who exhibit greater anxiety symptoms in all its expressions, are prone to more facial self–touching as is the case with those who have greater threat perception of COVID-19.

## 5. Conclusions

In conclusion, we point out that gloves seem to be a useful measure to reduce facial self–contacts. For all of the above reasons, we believe that it is necessary to establish educational actions and policies that will have an impact on the proper use of gloves to prevent contagion.

## Figures and Tables

**Table 1:** Descriptive analysis of the study variables.

Questionnaires	Mean	SD
STAI–S	18.26	13.91
BIPQ–5	29.28	11.39
Self–touching/minute Experimental situation 1	0.70	0.11
Self–touching/minute Experimental situation 2	0.52	0.14
Self–touching/minute Experimental situation 3	0.70	0.14
Heart rate/minute	75.26	11.36
Systolic blood pressure	135.15	9.45
Diastolic blood pressure	86.74	4.55

**Table 2:** Differences in Gender and previous psychological problems (anxiety and depression) for STAI–S, BIPQ–5 and self–contact in the different experimental situations.

Questionnaires	Gender		Previous Psychological Problems							
	Mean (SD) Man n = 62	Mean (SD) Woman n = 88	t	*p*	*d*	Mean (SD) Yes n = 26	Mean (SD) No n = 124	t	*p*	*d*
STAI–S	14.9 (11.88)	20.6 (14.8)	2.51	0.01 *	0.42	27.5 (19.1)	16.3 (11.7)	3.91	0.01 **	0.71
BIP–Q5	28.2 (11.66)	29.9 (11.2)	0.89	0.37	0.14	33.9 (10.6)	28.3 (11.3)	2.43	0.02 *	0.51
Self–contacts/minute ES 1	0.7 (0.1)	0.7 (0.1)	0.51	0.6	0.08	0.6 (0.1)	0.7 (0.1)	0.99	0.32	0.23
Self–contacts/minute ES 2	0.5 (0.1)	0.5 (0.1)	0.19	0.68	0.07	0.4 (0.1)	0.5 (0.1)	1.61	0.11	0.35
Self–contacts/minute ES 3	0.6 (0.1)	0.7 (0.1)	0.64	0.41	0.15	0.6 (0.1)	0.7 (0.1)	0.16	0.87	0.06
Heart rate/minute	75.9 (11.5)	74.8 (11.2)	0.58	0.55	0.09	79.6 (11.4)	74.3 (11.1)	2.18	0.02	0.47
Systolic blood pressure	134.5 (9.6)	135.5 (9.3)	0.63	0.52	0.11	137.3 (9.9)	134.7 (9.3)	1.22	0.22	0.26
Diastolic blood pressure	86.4 (4.5)	96.9 (4.5)	0.61	0.54	0.11	87.1 (4.8)	86.6 (4.5)	0.50	0.63	0.18

Note: ES = experimental situation. * Significant at the 0.05 level. ** Significant at the 0.01 level. ES 1 (no control measures), ES 2 (disposable gloves), ES 3 (reminder posters). t = t–value. *p* = probability value. d = Cohen’s d or effect size (small ≈ 0.2, medium ≈ 0.5 and high ≈ 0.8).

**Table 3:** Intercorrelations between variables studied (STAI–S, BIPQ–5, Self–contacts/minute Experimental situation 1–2–3, Heart rate/minute, Systolic blood pressure and Diastolic blood pressure) n = 150.

Questionnaires	1	2	3	4	5	6	7	8
STAI–S		0.188 *	0.220 **	0.045	0.191 *	0.454 **	0.328 **	0.136
BIPQ–5			–0.393 **	–0.466 **	–0.290 **	0.082	0.043	0.044
Self–contacts ES 1				0.738 **	0.824 **	0.274 **	0.188 *	0.214 **
Self–contacts ES 2					0.654 **	0.167 *	–0.003	0.025
Self–contacts ES 3						0.218 *	0.168 *	0.149
Heart rate							0.341 **	0.308 **
Systolic blood pressure								0.594 **
Diastolic blood pressure								

Note: ES = experimental situation. * Correlation is significant at the 0.05 level. ** Correlation is significant at the 0.01 level. ES 1 (no control measure‘s), ES 2 (disposable gloves), ES 3 (reminder posters).

**Table 4:** ANOVA measurement repeated for the self–contact variable in the different experimental situations.

Study	Self–Contacts ES 1	Self–Contacts ES 2	Self–Contacts ES 3	1–2	1–3	2–3
Facial Self–Contact	0.7 (0.1)	0.5 (0.1)	0.7 (0.1)	*p* < 0.01 ** IC [–2.1—1.6]	1 IC [–0.11–0.21]	*p* < 0.01 ** IC [–0.7 –1.2]

Note: ES = experimental situation. ** Significant at the 0.01 level l. ES 1 (no control measures), ES 2 (disposable gloves), ES 3 (reminder posters).

**Table 5:** Hierarchical Multiple Regression Prediction Self–contacts in ES 2 from health rate, STAI–S y BIPQ–5.

Variable	Frequency of Self–Contacts in ES 2					
	Model 1		Model 2		Model 3	
	B	β	B	β	B	β
Constant	5.49 **		7.59 **		7.83 **	
Heart rate	0.03 *	0.16	0.03 *	0.20	0.03 *	0.18
BIPQ–5			–0.09**	–0.48	–0.09 **	–0.49
STAI–S					0.01	0.05
						
R^2^	0.028		0.259		0.261	
F	4.223 *		25.709 **		17.229 **	
ΛR^2^	0.028		0.231		0.002	
ΛF	4.223 *		45.913 **		0.459	

Note. ES = experimental situation. ** Significant at the 0.01 level. ES 2 (disposable gloves). * Significant at the 0.05 level.

## Data Availability

The data that support the findings of this study are available on request from the corresponding author. The data are not publicly available due to privacy and ethical restrictions.

## Appendix A

The instruments are described in more detail in the online appendix. In addition, the administered online questionnaire is presented. (Link: https://github.com/mariajosegonzalez123/online-appendix.git (accessed on 29 June 2021))
